# The Influence of Increasing Concentrations of AMPD on the Efficacy of Its Penetration into a Model Skin Sebum Layer

**DOI:** 10.3390/pharmaceutics12121228

**Published:** 2020-12-18

**Authors:** Agnieszka Kostrzębska, Witold Musiał

**Affiliations:** Department of Physical Chemistry and Biophysics, Faculty of Pharmacy, Wroclaw Medical University, ul. Borowska 211A, 50-556 Wrocław, Poland; agnieszka.kostrzebska@umed.wroc.pl

**Keywords:** skin sebum, alcoholamines, 2-amino-2-methyl-1,3-propanediol, AMPD, acne

## Abstract

Alcoholamines are widely used as auxiliary substances in various topical preparations. Their impact on the components of skin sebum allows them to be used in preparations that cleanse the skin of sebum in hair follicles. We measured the effects of various concentrations of aqueous solutions of AMPD (2-amino-2-methyl-1,3-propanediol) on model skin sebum. The volume of reacted sebum was calculated using two methods: optical assessment of the interaction of alcoholamines with the components of model skin sebum and determination of the reacted volume of model skin sebum based on the measurements of changes in the pH of the AMPD solutions. Both methods showed that the most favorable AMPD concentration for model sebum penetration was approximately 1–2%. Lower values of alcoholamine caused premature exhaustion from the solution. Excessively high concentrations resulted in the formation of a dense layer of products hindering effective skin cleansing.

## 1. Introduction

Acne vulgaris is one of the most common skin diseases of adolescents and young adults. It is characterized as a sebum-producing sebaceous gland disorder. The main pathophysiology of acne involves abnormal exfoliation of epidermal cells, excessive sebum secretion, and the action of bacteria that colonize hair follicles [1]. As a result of the activity of bacteria of the human microbiome, such as *Propionibacterium acnes*, *P. granulosum*, *Staphylococcus epidermidis*, and *S. aureus*, there is the formation of sebaceous metabolic products including free fatty acids and other pro-inflammatory substances that cause hair follicle clogging [2,3,4,5].

For local acne treatment, antibacterial, hygiene, and care products are used most often. They work by removing the surface layer of lipids from the skin. Usually, however, they do not cleanse clogged sebaceous glands [6,7,8].

The proposed mechanism for cleansing sebaceous glands more thoroughly may be the binding of free fatty acids from sebum with alcoholamines to form amine soaps and easier removal of sebum from glands. [9,10,11]. Alcoholamines are characterized by emulsifying, thickening, foaming, and preserving properties. Their alkaline nature is used to regulate the pH of cosmetics. Many of them, including AMPD (2-amino-2-methyl-1,3-propanediol), MEA (monoethanolamine), DEA (diethanolamine), TEA (triethanoloamine), TRIS (tromethamine), MIPA (monoisopropanolamine), and TIPA (triisopropanolamine), are used in the cosmetics and pharmaceutical industries as excipients. They are used in the production of creams, ointments, shampoos, emulsions, lotions, and tonics. They are also widely used in pharmaceutical technology as auxiliary substances and derivatives for the production of medicines [12,13,14].

Previous studies have demonstrated the reactions between various alcoholamines and the components of the model sebum, leading to the formation of amine soap. The resulting soap binds water due to the presence of hydroxyl groups in the alcoholamine molecule. It stabilizes the O/W emulsion, increases the volume of sebum in the gland, and loosens it. This action facilitates the elimination of sebum from the gland. Together with sebum, P. acnes, which is responsible for acne inflammation, can be removed [9,10,11]. It is also possible to create alcoholamine complexes with various additives such as anionic polymers. These affect the prolongation of the action of alcoholamines and reduce the alkalinity of the system without affecting the reaction of alcoholamines with model sebum [10,11].

Based on previous studies and our observations of selected alcoholamines, we chose AMPD for further research. Its molecular weight is 105.14, pKa = 8.76 at 25 °C, it is soluble in water and alcohol [13]. We prepared aqueous solutions of AMPD with concentrations of 0.5% to 2.5%. The pH of these solutions did not exceed 11, which places AMPD in the middle of the alcoholamines selected for this study. Then we placed them on the model sebum layer, observing the changes for 4 h. We also carried out a 4-h measurement of changes in the pH value over the sebum in the AMPD solution. AMPD activity with respect to the components of model sebum was superior to that of other alcoholamines. AMPD penetrated well into the model sebum. The swelling of the ingredients over the sebum layer was also effective.

## 2. Materials and Methods

### 2.1. Reagents

The following reagents were used as model skin sebum components: triglycerides of animal origin (Cefarm, Warszawa, Poland), stearic acid (Sigma-Aldrich, Poznan, Poland), squalen (Sigma–Aldrich), cholesterol (Sigma–Aldrich), and lanolin (Cefarm). The following alcoholamines were selected as substances penetrating model skin sebum: AMPD (Sigma-Aldrich), TRIS (Sigma-Aldrich), DIPA (Sigma-Aldrich), and TIPA (Sigma-Aldrich). Demineralized and bi-distilled water were used to form aqueous solutions of alcoholamines.

### 2.2. Analytical Methods

#### 2.2.1. Composition and Preparation of Model Skin Sebum

The composition of a model skin sebum was based on the available literature. The skin sebum is a mixture of lipids that differ in chemical properties from biological membranes. Its composition includes triglycerides, squalene, cholesterol, and waxes [15,16,17]. Based on previous studies, model sebum was prepared to contain pork lard for triglycerides (34%), stearic acid as a free fatty acid (24%), lanolin as wax (26%), squalene (12%), and cholesterol (4%) [10,11]. This sebum formulation, developed by Kubis and Musiał, was recognized by Stefaniak and Harvey as containing important lipid classes with constituents at concentrations corresponding with human sebum including squalene, wax esters, triglycerides, free fatty acids, and free cholesterol [18]. The ingredients were melted and mixed in a water bath and left to solidify.

#### 2.2.2. Determination of Model Skin Sebum Density

The density of model skin sebum was determined using a pharmacopoeial method using a pycnometer [19]; 25% of the pycnometer volume was filled with molten model skin sebum and was then weighed after cooling to 20 °C. The volume was topped up with 20 °C water, and the sample was weighed again. Calculations were made based on Equation (1) [19]:

(1) $$d\;=\;\frac{m_{1}}{w\;+\;m_{1}-m_{2}}\;\times \;0.997\;+\;0.0012$$

where *d* is the density of model skin sebum, *m*_1_ is the mass of skin sebum, *w* is the mass of pycnometer with water and *m*_2_ is the mass of pycnometer with model skin sebum and water weighed at 20 °C. The value 0.997, given in the equation, is the density of water at 20 °C, and 0.0012 is a correction for air weighing. The density of model skin sebum used in the study was 0.842 mg/mm^3^. This value is similar to the sebum density of healthy people and people with acne (0.9 mg/mm^3^ ± 0.01) [20].

#### 2.2.3. Optical Assessment of the Interaction of Alcoholamines with the Components of Model Skin Sebum

To determine the volume of reacted sebum, the height of the reacted sebum was measured in polystyrene tubes of 16 mm in diameter. A 2 cm^3^ piece of sebum was placed in the tube, on which a 3 cm^3^ aqueous AMPD solution was applied. The course of the reaction was observed for 4 h at strictly defined intervals. The exact height of the reacted sebum layer was determined using digital macro photography, following the rule applied in the research of Kubis et al. [10,11]. We used a Fuji FinePix S6500FD digital camera (Fuji Photo Film Co., Ltd., Tokyo, Japan), 6.3 megapixels, with a 6.2–66.7 mm variable focal length lens, as well as an original program for digital image processing.

#### 2.2.4. Measurement of the Degree of Turbidity of the Evaluated Solution above the Sebum Layer

The turbidity of the evaluated solution over model sebum was measured using a single-beam spectrophotometer (Spekol 11, Carl Zeiss, Jena, Germany) using a TK1 attachment. Small particles can scatter short wavelengths of light more efficiently comparing to long wavelengths, which may result in disproportionately high measurement. To compensate for this effect during the measurements, we decided to use a light length close to the IR range (780–900 nm) [21]. The maximum wavelength, at which the measurements were carried out, was determined experimentally based on a series of diluted suspensions resulting from the highest concentration of AMPD with sebum components. This value was 850 nm. After four hours of observation, the solution with 0.5% AMPD showed the highest degree of turbidity in the solution. The turbidity of the evaluated solutions above the layer of model skin sebum decreased as the concentration of the AMPD increased.

#### 2.2.5. Measurement of Changes in the pH Value of AMPD Solutions over a Layer of Model Skin Sebum

Changes in the pH of the alcoholamine solution above the model skin sebum, associated with the reaction of amine soap formation, were measured using a Hanna Instruments pH302 (Hanna Instruments, Woonsocket, RI, USA) with a suitable computer program. pH measurements were taken at one-minute intervals for four hours. We applied 3 g of AMPD solutions to a 2 cm thick model skin sebum layer placed in a tube with a diameter of 16 mm. The tubes were protected against air by paraffin film. The measurements used a glass electrode OSH 10-10 adapted for measurements in small volumes of liquid. During the pH measurement, the electrode was placed high in the AMPD solution so as not to touch the sebum layer.

### 2.3. Calculation Methods

#### 2.3.1. Calculation of the Penetrated and the Increased Volume of the Sebum Using a Proprietary Computer Program

**Figure 1:**
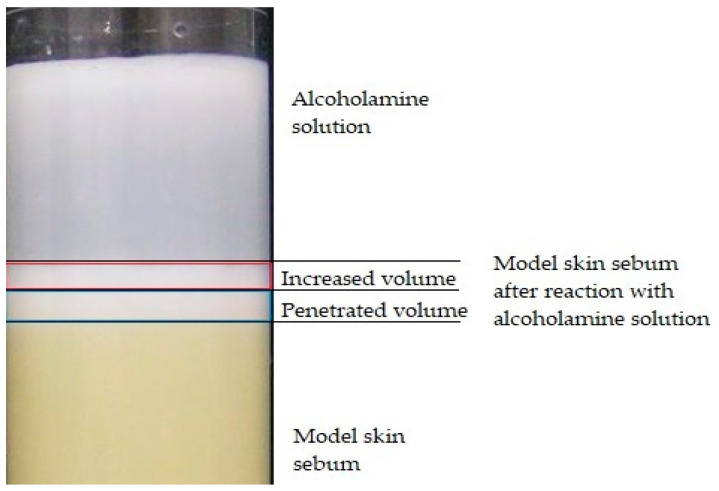
Measurement of sebum volume using the optical method and computer program for calculations.

The image height of the sebum column was measured with an accuracy of 0.02 mm with a fifty-fold magnification of the image on the monitor. Calculations were made based on the number of pixels corresponding to 1 mm in length. The optical method, modified in the Department of Drug Form Technology, Faculty of Pharmacy, Wroclaw Medical University, was adapted to measure the height of the sebum column [9,10]. The method of observation and calculations are presented in Figure 1.

Alcoholamine reacts with the components of model skin sebum, e.g., stearic acid, and amine soap is formed. The reaction is manifested by the formation of a bright, reacted layer above the intact sebum layer. The resulting soap, by binding water, loosens the remaining sebum components of the sebum layer, unreacted with AMPD, facilitating their detachment and transfer to the solution. Both the penetrated volume and the increased volume of sebum are composed of the resulting soap and loosened sebum. Measurements were taken of the height of the sebum column that reacted with alcoholamine―the etching of the sebum layer was ascribed as the penetration depth, and this description was applied consequently in the study―and the height of the reaction products obtained (height of increase) beyond the initial level. On this basis, we calculated the volume of reacted sebum (penetrated volume) and the volume of sebum raised above the initial level (increased volume). Calculations were made based on Equation (2):

*V = π·r^2^·h* (2)

where *V* is the volume (penetrated or increased), *h* is the height of reacted sebum, and *r* is the tube radius. The reaction for each concentration of AMPD with sebum was performed four times.

#### 2.3.2. Calculation of the Volume of Reacted Sebum Based on Measured Changes in the pH Value of a Given Solution.

The reaction of alcoholamine with stearic acid contained in the model skin sebum runs according to Equation (3) [22]:

*R_1_–COOH + R_2_–NH_2_ (aq) → R1–COO–[NH_3_^+^–R_2_] (aq)* (3)

It is the reaction of an undissociated weak base molecule present in solution with a weak acid molecule that is a component of the model skin sebum, resulting in the formation of an amine soap. According to observations made by Zhu et al. on similar soap composition i.e., TEA stearate, it should be assumed that the resulting amine soap slowly hydrolyses over time [23]. However, this process is slow and lengthy, thus the changes were observed after several dozen hours. In the course of research by Zhu et al., it was shown that stearic acid formed during hydrolysis, which is a weak acid insoluble in water, was deposited in the form of plaques, while the weak base remained in the aqueous solution. Therefore, the presence of stearic acid in the form of dispersed plaques should not have a significant influence on changes in the pH value of the solution above the sebum [23]. Our tests were carried out in a much shorter period of time, not exceeding 4 h, and also in stable environmental conditions, assuming the hydrolysis process to an insignificant degree.

To calculate the loss of alcoholamine from the solution during the reaction with the components of model skin sebum, we used Equation (4) to calculate the pH of the weak base solution [24,25]:

(4) $$pH={pK}_{w}-\frac{1}{2}\;({pK}_{b}\;-\;{logC}_{b})$$

where *pH* is the measured AMPD solution pH value, *pK_w_* is the exponent of the ionic product of water, *pK_b_* is the exponent of the base dissociation constant, and *C_b_* is the total molar concentration of base (mol/dm^3^).

After transformation, the molar concentration was calculated (Equation (5)):

*C_b_ = 10 ^2pH − 2pKw + pKb^* (5)

Knowing the molar concentration of the undissociated alcoholamine in the solution, its mass was calculated according to Equation (6):

*m_z_ = C_b_·M·V_r_* (6)

where *m_z_* is the mass of the undissociated alcoholamine (g), *M* is the AMPD molar mass (g/mol), and *Vr* is the volume of AMPD solution (dm^3^).

The mass of reacting alcoholamine was calculated according to Equation (7):

*m_zp_ = m_z −_ m_zn_* (7)

where *m_zp_* is the mass of reacted alcoholamine (g), *m_z_* is the mass of unreacted alcoholamine in the time t_0_ (g), and *m_zn_* is the mass od unreacted alcoholamine in the time t_n_ (g).

The mass of stearic acid that reacted with the alcoholamine was calculated according to Equation (8):

(8) $$m_{kp}=\;\frac{105.14\cdot mzp}{284.48}\;$$

where *m_kp_* is the mass of reacted stearic acid (g), 105.14 is the AMPD molar mass (g/mol), and 284.48 is the stearic acid molar mass (g/mol).

The mass of reacted sebum was calculated knowing the percentage of stearic acid contained within it (24%). The volume of reacted sebum was calculated according to Equation (9):

(9) $$V_{s}=\frac{ms}{ds}\;$$

where *m_s_* is the mass of reacted sebum (g) and *d_s_* is the density of model skin sebum(g/dm^3^).

## 3. Results

### 3.1. Initial Assessment of Selected Alcoholamines

Four alcoholamines were selected for preliminary tests: AMPD, TRIS, TIPA, and DIPA. 0.1M aqueous solutions were made. These alcoholamines are used as auxiliary or active substances in medical or cosmetic preparations [12,13,14]. The choices were also based on earlier studies of the interaction of alcoholamines with sebum [9,10]. DIPA was selected as a representative of secondary alcoholamine to assess its activity against model skin sebum.

TIPA, AMPD, and TRIS did not exceed pH 10.5. The alcoholamines with the lowest pH were selected. Preliminary tests of reaction with model skin sebum were carried out for all alcoholamines (Table 1).

TIPA had the best penetration volume. Slightly less sebum volume reacted under the influence of AMPD, and the smallest reacted under the influence of TRIS. TIPA had the lowest value of increased volume, which may suggest the formation of a dense layer of products hindering the effective removal of sebum. For this reason, AMPD was selected for further studies.

### 3.2. The Effect of AMPD with the Model Skin Sebum

**Figure 2:**
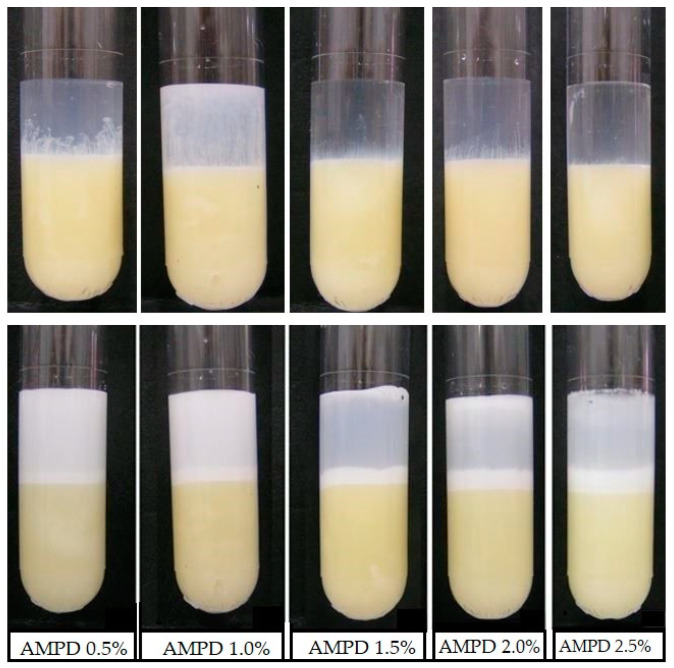
The reaction of AMPD (2-amino-2-methyl-1,3-propanediol) solutions of various concentrations with the components of model skin sebum. Line 1—At time = 0, line 2—After four hours of observation.

AMPD aqueous solutions of various concentrations interacted with the components of model skin sebum. We carried out 4-h observations of changes taking place on the sebum surface after placing the alcoholamine solution over it. Alcoholamine reacted with the ingredients of the model skin sebum such as stearic acid to form amine soap. The products of this reaction were visible in the form of a bright, reacted layer forming on the sebum layer. Alcoholamine penetrated deeply into the sebum layer. Simultaneously, the resulting layer of soap increased above the initial level of sebum. The volume of reacted sebum depended on the concentration of the AMPD solution used. At the lowest concentration, i.e., 0.5%, the smallest layer of reacted sebum and the highest turbidity of the solution above the sebum were observed. The penetration layer volume increased with increasing AMPD concentration. Simultaneously, the turbidity of the solution decreased. These changes are presented in Figure 2.

The turbidity of the alcoholamine solution above the model skin sebum during observation depended on the concentration of the AMPD solution used. Low concentration solutions had a high degree of turbidity. As the alcoholamine concentration increased, the turbidity of the solution decreased (Table 2); the initial turbidity in all cases had the zero value.

### 3.3. Determination of Penetrated and Increased Sebum Volume Based on Observations and Calculations Made Using a Computer Program

**Figure 3:**
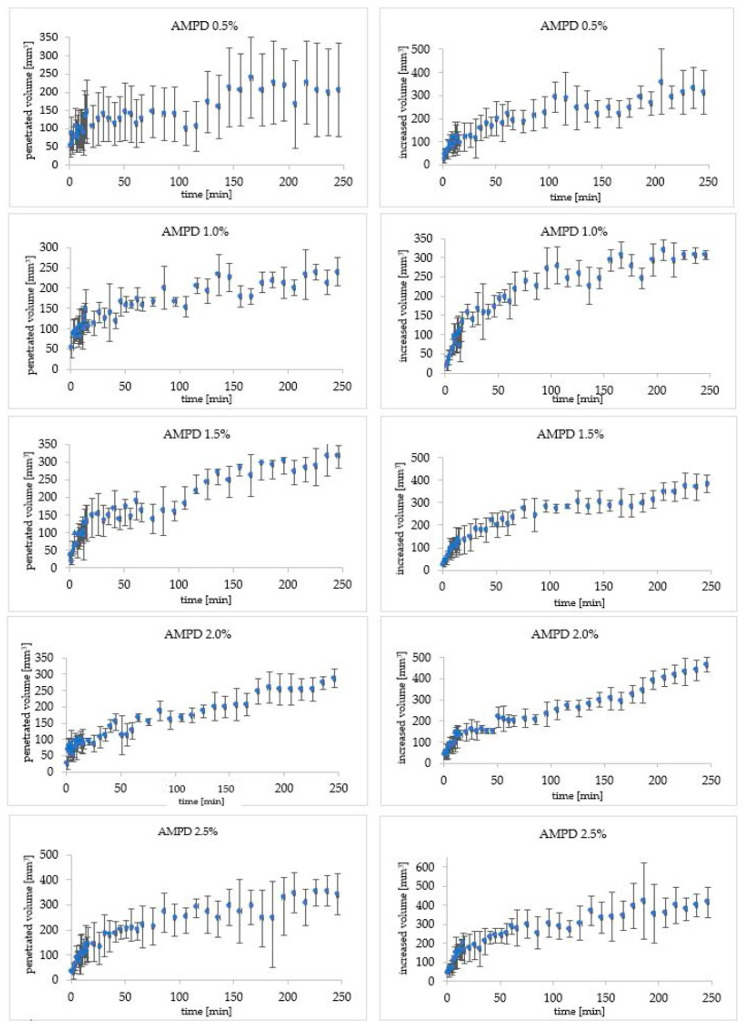
The course of changes in the increased and penetrated volume of model skin sebum under the influence of AMPD solutions for individual concentrations of AMPD solutions with the presentation of standard deviations (SD).

**Figure 4:**
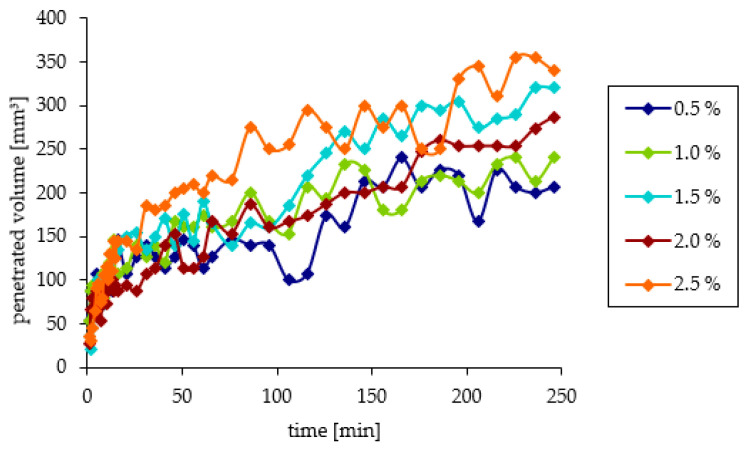
The course of changes in the penetrated volume of the model skin sebum under the influence of AMPD solutions with various concentrations within 240 min.

The initial course of the reaction of the alcoholamine with the sebum layer was similar for all concentrations used. Over time, differences in the effects of AMPD solutions with various concentrations on sebum were observed. After four hours of observation, the penetrated volume of the model skin sebum for the lowest concentration of AMPD solution had the lowest value and vice versa. The penetrated volumes of model skin sebum were close to each other, what was confirmed via the evaluation of the standard deviations (SD) (Figure 3), however, the observed tendency indicated more intense interaction in the case of high concentration of the alcoholamine, comparing to the low concentration of alcoholamine (Figure 4).

**Figure 5:**
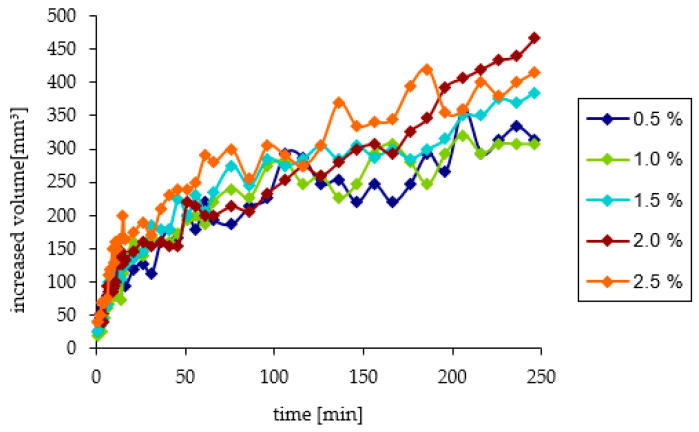
The course of changes in the increased volume of model skin sebum over the initial level under the influence of AMPD solutions with various concentrations within 240 min.

Changes in the increased volume of reacted sebum above the initial level were slightly different and un-significant in the terms of standard deviations measures (Figure 3). During the first 16 min, the volume increased at a similar pace. After 216 min of observation, for solutions with lower concentrations, i.e., 0.5% and 1.0%, the inhibition of volume growth was observed and its value remained constant until the end of the observation. For solutions with higher concentrations, the reaction continued up to 240 min. The increased sebum volume for the highest concentration of AMPD solution reached a value similar to the 1.5% AMPD solution. The highest value of increased volume appeared for the 2.0% AMPD solution (Figure 5). The reaction for each concentration of AMPD with sebum was performed four times.

### 3.4. Changes in the pH Value in the AMPD Aqueous Solutions above the Layer of Model Skin Sebum

**Figure 6:**
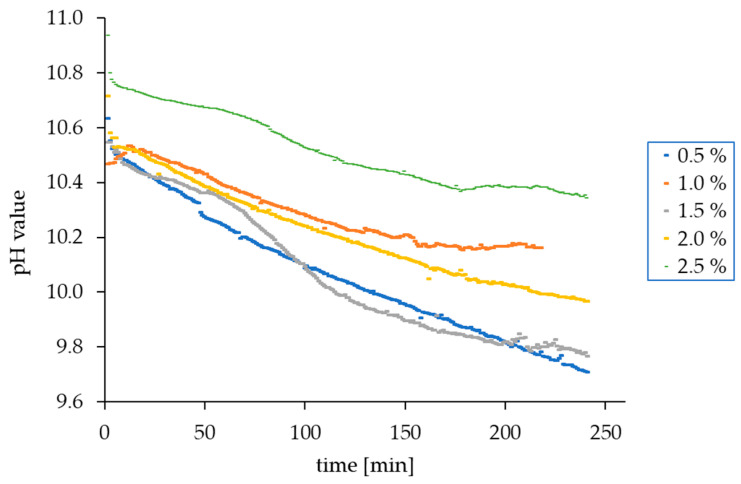
A decrease in the pH value of AMPD solutions with various concentrations over the model skin sebum within 240 min.

The pH values of AMPD solutions varied depending on the concentration of alcoholamine in the range of 10.47–10.94. After placing the AMPD solution over a layer of model skin sebum, decreases in these values were observed over four consecutive hours. During the first few minutes of observation, the rate of change was quite high, then it slowed down and stabilized, gradually decreasing to 9.7–10.3, depending on the concentration of alcoholamine (Figure 6). The samples of 1.0% and 1.5% AMPD concentration deviated from the patterns, observed in the case of the other samples. This nuisance may be attributed to the intense initial flotation of reacted sebum particles (Figure 2. 1.0% and 1.5% AMPD, upper row), which presumably hindered the flux of hydrogen ions via the electrode membrane during the pH assay in the initial phase of the process. The assay was terminated after 4 h presumed for a practical application time of such composition in the future drug form.

### 3.5. Determination of Reacted Volume of Model Skin Sebum Based on the Measurements of Changes in pH of the AMPD Solutions

**Figure 7:**
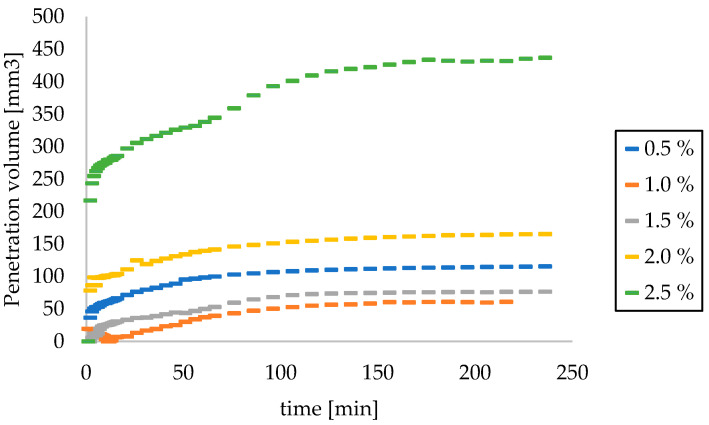
The reacted volume of the model skin sebum was determined based on changes in the pH of AMPD solutions.

The volume of model skin sebum reacted with AMPD solutions was calculated based on measurements of the pH value of alcoholamine solutions. During the first few minutes, the reaction proceeded quickly and then decelerated and stabilized toward the end of the observation. The solution with the highest concentration had the largest penetration volume. The remaining AMPD solutions reacted similarly (Figure 7).

**Figure 8:**
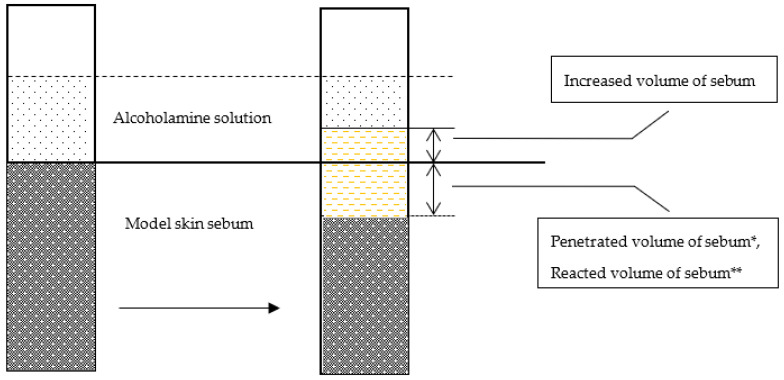
Increased and penetrated volume of model skin sebum calculated using the optical method, the computer program, and the reacted volume of model skin sebum based on the measurements of changes in the pH of the AMPD solution, modified according to a previous study [9]. * Penetrated volume of the sebum was determined via direct optical observation. ** Reacted volume of the sebum was calculated from acidity-alkalinity data of the reacting system. The composition of penetrated and increased volume of sebum is of the same origin—sebum components reacted with the AMPD and loosened with water.

The volume of sebum that reacted with the alcoholamine solution was determined practically based on the observations of changes and calculations made using a computer program. The volume of reacted sebum was also determined theoretically based on the measurements of changes in the pH value of the alcoholamine solution over the layer of model sebum (Figure 8).

## 4. Discussion

As shown in previous studies, various alcoholamines react with the components of model skin sebum, including stearic acid, to form amine soaps. The products of this reaction are visible as bright, reacted layers formed on the intact layer of sebum. Penetration of the alcoholamine into the depth is accompanied by the rising of the resulting soap layer above the initial level of sebum. This is because the resulting soap with emulsifying properties can bind large amounts of water, which softens the sebum layer [9,10,11]. Saponification and softening of sebum with alcoholamines can be an effective way to deeply cleanse skin with acne. As shown in Table 1, due to the positive course of penetration deep into the sebum and the rise of the resulting products above the sebum layer, 2-amino-2-methyl-1,3-propanediol was selected for the study. The reaction of AMPD with components of model skin sebum has already been observed in earlier studies [9]. The pH value of AMPD aqueous solutions is approximately 10.5 and is not as high as that of some of the alcoholamines considered. This compound is used in the cosmetics and pharmaceutical industries as an auxiliary substance; therefore, it is an interesting object for further research in terms of its use as a substance that facilitates skin cleansing [13].

The concentration of the AMPD aqueous solution has a significant effect on the course of the reaction. During the first several minutes of observation, there was no significant difference in the volume of the product layer between individual concentrations. The increased volume of sebum above the initial level in the 21st-minute observation for a 0.5% solution was approximately 120 mm^3^ and, for a 2.5% solution, it was approximately 175 mm^3^. The reason may be the presence of a saturated alcoholamine solution above the sebum layer and a small reaction surface. Over time, the differences in the increased volume above the level of sebum became increasingly visible. For solutions with lower concentrations, a small increased volume of sebum was observed, which may be associated with the low concentration of the alcoholamine used, and rapid depletion of its contents in the solution. For the 0.5% solution, the increased volume after four hours of observation was approximately 313 mm^3^ and, for the 2.5% solution, it was approximately 415 mm^3^. The situation is similar for the penetrated volume. For the 0.5% solution, the penetrated volume of the model sebum at the 21st minute was approximately 107 mm^3^. For the 2.5% solution, it was 145 mm^3^. After four hours of observation, the penetrated volume for the 0.5% solution was 207 mm^3^ and, for the 2.5% solution, it was 340 mm^3^. A similar course of the reaction of alcoholamine with the components of model sebum was observed in previous studies using triethanolamine neutralized with anionic polymers [10,11].

For solutions with low concentrations of AMPD, where the layer of uplifted products had a small thickness, high turbidity of the solution above the reaction layer was visible, suggesting great ease of movement of the particles of reacted sebum. For solutions with higher concentrations, the reaction product layer is thicker and the turbidity of the solution is smaller; the reaction products remained near the reaction surface, in contrast to solutions with lower concentrations, in which the reaction products freely moved within the solution. This may be due to the high density of the products obtained and the low porosity of the product layer. These features may hinder the detachment of reacted sebum particles and their diffusion into the solution. Differences in turbidity for solutions with various concentrations are shown in Table 2.

During the reaction of the aqueous AMPD solution with the components of the model sebum, a gradual decrease in the pH value of the solution was observed. This was associated with a neutralization reaction between stearic acid contained in sebum and alcoholamine. The equation applied in our calculations to approximate the AMPD level based on pH has illustrative value, as the ionic strength was not included in the evaluations, due to the complex composition of the reactants. However, in our opinion, the obtained AMPD levels may be informative for the processes observed in the studied environment. The obtained pH results may vary from the equilibrium state, due to the dynamics of the system, where the AMPD molecules overcome the specific diffusion path. However, the presented results reflect both the applicative potential of the AMPD solution, as well as the local pH changes in the course of the etching reaction. The decrease in the pH value of the alcoholamine solution is initially sharp and slows down over time. As Figure 6 shows, for the first minutes, the value decreases quite quickly and then decelerates until the end of the observation. This may be due to the depletion of alcoholamine in solution and the resulting inhibition of the reaction of the alcoholamine with the components of model skin sebum. This particularly applies to solutions with lower concentrations. In the case of solutions with higher concentrations, inhibition may be caused by the formation of a thick layer of high-density reaction products that may cause difficulties in the access of alcoholamine to sebum.

Comparison of actual, observed changes in sebum volume with changes calculated based on pH value allows the identification of additional parameters affecting penetration. Changes in the density of reaction products relative to the density of sebum and an aqueous solution affect the mutual reactivity of alcoholamine and acid. An additional factor may be the formation of local soap aggregates and their chaotic, accidental detachment from the surface of saponified sebum.

The optical method of observing the changes taking place over the sebum should take into account the fact that the emerging layer of reagents, in addition to the soap, also includes soap-bound water. The process of water-binding by the resulting amine soap leads to a continuous, slow increase in volume and loosening of the reacted sebum layer despite the depletion of reagents in the solution above the sebum. Table 3 shows the values of penetrated volume and reacted volume after 216 min.

The small differences between the values for the 0.5% AMPD solution may be because the alcoholamine may have been depleted in the solution before the end of observation. The difference for a 2.5% solution may be due to the appearance of a hard-permeable layer of products at the interface of sebum and AMPD solution. Larger differences in volume values for intermediate solutions may be because the resulting soap loosens the sebum layer by binding water.

The study presents the possibility of determining the volume of model skin sebum reacted with alcoholamine using two different methods. Both methods (the optical-based method based on observations of changes over the layer of model sebum and the method based on measurements of changes in pH value) show the rapid initial course of the reaction of alcoholamine with the components of model skin sebum and then the inhibition of the reaction.

AMPD is commonly used in various cosmetic products. As presented in the report by Burnett et al. regarding the safety of using AMP and AMPD, these substances are used as auxiliary substances in make-up products, skincare, and hygiene cosmetics. The concentration of AMPD in these products ranges from 0.1–2%, and it is usually used as a substance that stabilizes the pH of the product [26]. According to the results of our study, the use of AMPD in agents for skin cleansing, including skin with acne, in concentrations close to 1–2% can be considered for its ability to react with the components of skin sebum. This value can provide more effective skin cleansing of the remaining sebum. Using low percentages (0.5% and less) may be insufficient for effective skin cleansing due to the rapid depletion of alcoholamine from the product. Excessively high concentrations (over 2%) may cause the formation of a dense layer of reaction products on the surface of the sebum that prevents better penetration into the hair follicles and thus weaker skin cleansing.

It should be noted that the amine soap resulting from the reaction of AMPD solution with stearic acid is a salt of a weak base and a weak acid. Assuming that the hydrolysis of salts of weak bases and weak acids occurs according to Equation (10)

*R_1_–COO–[NH_3_^+^–R_2_] → R_1_–COOH + R_2_–NH_2_* (10)

as a result of this process, a weak base and weak acid are formed, which are practically undissociated electrolytes. The equation for the constant hydrolysis of salts of a weak acid and weak base takes the form of Equation (11) [27]:

(11) $$K_{h}=\frac{K_{w}}{K_{a}\cdot K_{b}}\;$$

assuming that for AMPD, the *K_b_* = 6.309 × 10^−6^; for stearic acid, *K_a_* = 1.778 × 10^−5^, and *K_w_* = 10^−14^.

The value of *K_h_* = 8.912 × 10^−5^ and the pH of the salt solution of a weak base and a weak acid calculated from Equation (12) [28] is 6.775.

(12) $$pH=\frac{1}{2}({pK}_{w}\;+\;{pK}_{a}-{pK}_{b})$$

The slightly acidic reaction is because the dissociation constant of the weak acid has a slightly higher value than the base dissociation constant.

Based on these theoretical considerations, it can be assumed that the hydrolysis of the resulting soap does not occur or takes place to a very small extent without affecting the pH value of the solution above the layer of model skin sebum. However, Zhu et al. demonstrated the hydrolysis and decay of soap by conducting a series of studies on the behavior of acid soap triethanolamine stearate under varied temperature conditions and in a long observation period [23]. Researchers prepared TEA stearate at a temperature of 80 °C, and then the formed soap was cooled to ambient temperature and observed for a period of 90 days. Changes and gradual decomposition of soap were observed in the given time interval. Nevertheless, testing of the stability of the presented AMPD stearate system and its possible breakdown should be further considered. This issue is important for estimating the course of action for the proposed formulation with AMPD as a skin cleansing agent against excessive sebum presence.

## 5. Conclusions

The effect of alcoholamine on the components of model skin sebum has been demonstrated. The most favorable AMPD concentrations for an effective reaction with the components of model skin sebum oscillate between 1.0% and 2.0%. Solutions with a concentration of 0.5% were insufficient and solutions with a concentration of approximately 2.5% caused the formation of a thick layer of products inhibiting the saponification reaction of the ingredients.

The presented study is an introduction to further research aimed at demonstrating whether there is a possibility of an effective combination of alcoholamines with antibacterial agents and creating a dual-action preparation-cleansing the skin and hair follicles and antibacterial effect, e.g., in the form of an ointment. Then prolonged-term application to the skin could take place. Appropriate selection of concentration of AMPD used in the designed product will enable effective use of its saponifying properties and avoid the phenomena observed in this study like the appearance of a layer that hinders the saponification or low AMPD concentration.

During the study, two methods were used to determine the course of the reaction of aqueous AMPD solutions with components of model sebum. Both showed similar courses of the reaction and congruous changes occurring in the layer of model sebum.

## Figures and Tables

**Table 1:** Alcoholamine comparison. Values of penetrated volume, increased volume, and pH after four hours of observation.

Alcoholamine	AMPD	TRIS	DIPA	TIPA
Penetrated volume of model skin sebum, *n* = 1 (mm^3^)	397	332	357	427
Increased volume of model skin sebum over initial level, *n* = 1 (mm^3^)	651	813	692	521
pH value at the beginning of the reaction, *n* = 1 *	10.468 ± 0.01	10.444 ± 0.01	10.708 ± 0.01	10.063 ± 0.01
pH value after four hours, *n* = 1 *	10.146 ± 0.01	9.182 ± 0.01	9.853 ± 0.01	8.857 ± 0.01

* the variability due to the device accuracy.

**Table 2:** Changes in the turbidity of AMPD solutions with various concentrations over model skin sebum (*n* = 1).

Time [h]	AMPD 0.5%	AMPD 1.0%	AMPD 1.5%	AMPD 2.0%
1	0.291	0.200	0.146	0.036
2	0.423	0.235	0.214	0.058
3	0.476	0.269	0.294	0.114
4	0.532	0.430	0.351	0.178

**Table 3:** Values of penetrated volume and reacted volume after 216 min.

Volume of Reacted Sebum Calculated by Two Methods in 216 min	AMPD 0.5%	AMPD 1.0%	AMPD 1.5%	AMPD 2.0%	AMPD 2.5%
Penetrated volume of model skin sebum based on observations, *n* = 4 (mm^3)^	226.67 (SD = 112.6)	233.33 (SD = 61.91)	285 (SD = 30)	253.33 (SD = 34.16)	310.00 (SD = 52.91)
Calculated volume of the reacted model skin sebum determined based on measurements of AMPD solution pH changes, *n* = 1 ** (mm^3^)	115.1 (SD = 0.144)	61.02 (SD = 0.869)	76.46 (SD = 0.162)	164.88 (SD = 0.408)	31.77 (SD = 2.439)
difference	111.57	172.31	208.54	88.45	121.77 *

* the negative number confirms the production of rigid plug in the place of the reaction assuming that the application of higher concentration of the AMPD has limited cleansing efficacy, ** the variability was calculated due to the device accuracy.
